# Appointment

**Published:** 1853-11

**Authors:** 


*Appointment.*—Prof. Millington, formerly of William and Mary College, has been appointed Professor of Chemistry in the Memphis Medical College. Prof. M. takes up his residence at Memphis. S. B. H.

